# Chinese burdens and trends of diabetic retinopathy 1990–2021 and 15 years forecast: results from the Global Burden of Disease Study 2021

**DOI:** 10.3389/fendo.2025.1573581

**Published:** 2025-05-13

**Authors:** Chun Jiang, Xiuhui He, Yingying Zhu, Liming Tao

**Affiliations:** ^1^ Department of Ophthalmology, The Second Affiliated Hospital of Anhui Medical University, Hefei, Anhui, China; ^2^ Department of Ophthalmology, Lu’an Hospital Affiliated to Anhui Medical University, Lu’an, Anhui, China

**Keywords:** age-standardized rates, blindness, vision loss, diabetic retinopathy, years lived with disability

## Abstract

**Objectives:**

To assess the Chinese burden of diabetic retinopathy (DR) between 1990 and 2021, considering variations by year, age, and gender, as well as to forecast the trends over the next 15 years.

**Methods:**

We evaluated the burden of DR in China based on data from the GBD 2021, examining prevalence, years lived with disability (YLDs), age-standardized YLDs rates (ASYR), and age-standardized prevalence rates (ASPR) over the period 1990 to 2021. Furthermore, we utilized joinpoint analysis and the Bayesian age-period-cohort model to explore the epidemiological patterns of the disease and forecast its impact for the years 2022 to 2036.

**Results:**

In 2021, the number of YLDs and prevalence attributed to DR were 86,317 (95% UI: 56,595 to 125,565) and 1.37 million (95% UI: 1.04 to 1.78), respectively. Over the period 1990 to 2021, the AAPC of ASYR and ASPR for DR rose by 0.71 (95% CI: 0.28, 1.14) and 0.43 (95% CI: 0.20, 0.66) in China. Older adults and women experienced a greater burden. Aging and demographic changes are key risk factors for DR, and future trends suggest a decrease in ASYR and ASPR.

**Conclusion:**

The Chinese burden of DR has increased during the years 1990 to 2021. Despite the anticipated decline in the burden of DR between 2022 and 2036, the importance of bolstering efforts in DR prevention and control should not be underestimated.

## Introduction

With the global population steadily increasing, the prevalence of diabetes has surged over the past few decades, with the number of affected individuals rising at an alarming rate. By 2050, the global diabetic population is projected to reach 1.3 billion (1). Diabetic retinopathy (DR), recognized as a principal microvascular disease linked to diabetes, is the primary cause of visual loss (VL) among diabetic patients and a major contributor to vision loss in individuals aged 20 to 74 years (2). Research indicates that approximately 34.6% of diabetic patients develop DR, with 10.2% experiencing varying levels of vision impairment (3). By 2045, the global DR population is estimated to reach 160 million, posing significant challenges to quality of life and exacerbating the global healthcare burden (4).

Standing as the planet’s second economy and the largest developing nation, China not only hosts the world’s largest population, but also faces the challenge of having the largest number of people with diabetes. By 2045, the number of individuals with diabetes in China is projected to reach 174 million, with approximately 16.3% developing DR and 3.2% experiencing vision-threatening conditions (5). DR has emerged as a leading cause of vision loss among individuals aged 50 and older (6). Beyond its direct health impact, vision impairment profoundly diminishes patients’ quality of life and places a substantial socioeconomic burden on society. According to the GBD study, vision loss ranks third among all disease-related disabilities (7). Studies demonstrate that the prevalence of DR in China has been steadily rising, with its associated burden intensifying due to population growth and societal changes. These trends underscore the urgent need for effective DR prevention strategies (8).

Using the latest GBD data from 2021, this study systematically analyzed the spatial and temporal patterns of DR in China from 1990 to 2021, with a focus on age- and sex-specific burdens, as well as trends in DR prevalence and YLDs. Additionally, we projected the Chinese disease burden of DR over the next 15 years. Our findings not only build upon previous studies but also offer critical insights for formulating targeted strategies to prevent and manage DR in China.

## Methods

### Data sources and descriptive analysis

The present investigation employed the newest findings from the GBD conducted in 2021 (http://ghdx.healthdata.org). The GBD uses a meta-regression framework to address data limitations and variability, providing several metrics for evaluating disease burden, such as prevalence and YLDs. In accordance with the criteria established by the GBD analysis, vision loss associated with DM is attributed solely to DR (9). We collected Chinese data from the GBD 2021 dataset regarding YLDs and the prevalence of DR, covering the period spanning 1990 to 2021. In the database, we extracted data on the etiology of DR encompassing both type 1 diabetes mellitus (T1DM) and type 2 diabetes mellitus (T2DM). The degree of visual impairment was also categorized as moderate vision loss (MVL), severe vision loss (SVL), and blindness. As per the Snellen chart, a visual acuity (VA) of <6/18 but ≥6/60 was classified as MVL, SVL as VA <6/60 but ≥3/60, and blindness as VA <3/60 or a visual field around central fixation <10% (8). Under the purview of the GBD research, the disease burden associated with DR was assessed beginning at 20 years of age.

### Joinpoint regression analysis

To assess trends in the disease burden associated with DR over time, we utilized the joinpoint regression model, which consists of a series of linear statistical models. This model estimates shifts in disease rates using the least squares method, overcoming the limitations of conventional linear trend analyses (10). For this analysis, we employed Joinpoint software (version 4.9.1.0). We additionally computed the average annual percentage change (AAPC) and evaluated the significance of variations across different segments by comparing the AAPC with zero.

### Age-period-cohort analysis

Age-period-cohort models, frequently used in sociology and epidemiology, were applied to depict the changing trends in DR prevalence over the years 1990 to 2021. We conducted an APC analysis using a tool developed by Rosenberg and colleagues (11). For model fitting, age groups were divided into categories such as 20–24, 25–29, 30–34, and so on. For each 5-year period (1992–1996, 1997–2001…2017–2021), the total number of cases and cumulative prevalence rates across age groups were calculated. Model fitting was conducted using the Epi package in R (version 4.4.1).

### Decomposition analysis

We conducted a decomposition analysis to break down the burden of DR into components such as population age, population size, and prevalence (12). This study sought to deepen the understanding of how various demographic and disease-related factors affect the burden of DR. By separating the burden into epidemiological changes, population growth, and aging, we were able to more accurately assess the impact of age distribution, shifts in population size, and epidemiological factors on the DR burden in China.

### Bayesian age–period–cohort analysis

We performed Bayesian age–period–cohort (BAPC) analysis in R (version 4.4.1) using the BAPC and INLA software packages (13), enabling us to project ASYR and ASPR trends by gender for DR in China from 2022 to 2036.

## Results

### Descriptive analysis

In 2021, the total YLDs and cases attributed to DR in China reached 86,317 (95% UI: 56,595 to 125,565) and 1.37 million (95% UI: 1.04 to 1.78), respectively. The ASYR ascended from 3.21 (95% UI: 2.14 to 4.77) per 100,000 people in 1990 to 4.04 (95% UI: 2.67 to 5.88) per 100,000 in 2021. Similarly, the ASPR for DR rose from 55.59 (95% UI: 42.4 to 72.96) to 63.75 (95% UI: 49.13 to 82.08) per 100,000 people during the same period (**Table 1**, **Supplementary Table 1**). The prevalence of T2DM among all DR patients with visual impairment amounted to 1.36 million (95% UI: 1.03 to 1.76), which is about one hundred times more than T1DM. Among the three types of visual impairment, MVL had the highest prevalence, with 1.08 million (95% UI: 0.77 to 1.45) cases, followed by blindness, while SVL ranked lowest (**Table 1**).

**Table 1:** All-age cases and age-standardized YLDs, and prevalence rates in 2021 for DR in China.

Measure	Rei	All-ages cases (95%UI)			Age-standardized rates per 100000 people (95%UI)		
		T1DM	T2DM	DM	T1DM	T2DM	DM
YLDs	MVL	289 (145,534)	32260 (17569,55664)	32550 (17736,56177)	0.01 (0.01,0.02)	1.49 (0.83,2.56)	1.5 (0.83,2.58)
	SVL	123 (69,214)	13380 (7761,22302)	13504 (7835,22512)	0.01 (0,0.01)	0.64 (0.37,1.04)	0.64 (0.38,1.05)
	B	395 (234,652)	39868 (25448,61483)	40263 (25688,62051)	0.02 (0.01,0.03)	1.88 (1.21,2.9)	1.9 (1.22,2.93)
	BVL	808 (503,1298)	85509 (56096,124336)	86317 (56595,125565)	0.04 (0.02,0.06)	4.01 (2.65,5.82)	4.04 (2.67,5.88)
Prevalence	MVL	9355 (6189,13874)	1067063 (763418,1434422)	1076418 (770742,1446629)	0.43 (0.29,0.63)	49.25 (35.86,65.78)	49.68 (36.17,66.26)
	SVL	670 (423,1006)	75037 (50280,105921)	75707 (50761,106830)	0.03 (0.02,0.05)	3.58 (2.41,5)	3.61 (2.43,5.04)
	B	2108 (1406,3060)	219243 (160579,294722)	221351 (162103,297430)	0.1 (0.07,0.15)	10.35 (7.6,13.69)	10.45 (7.68,13.82)
	BVL	12133 (8477,17093)	1361343 (1031914,1763840)	1373476 (1040302,1779196)	0.57 (0.4,0.79)	63.18 (48.73,81.37)	63.75 (49.13,82.08)

“Rei” refers to the classification of burden type in the GBD framework, indicating whether the estimate pertains to a risk factor, etiology, impairment, or injury (source: IHME REI metadata); YLDs, years lived with disability; MVL, moderate vision loss; SVL, severe vision loss; B, blindness; BVL, blindness and vision loss; UI, uncertainty interval.

**Figure 1:**
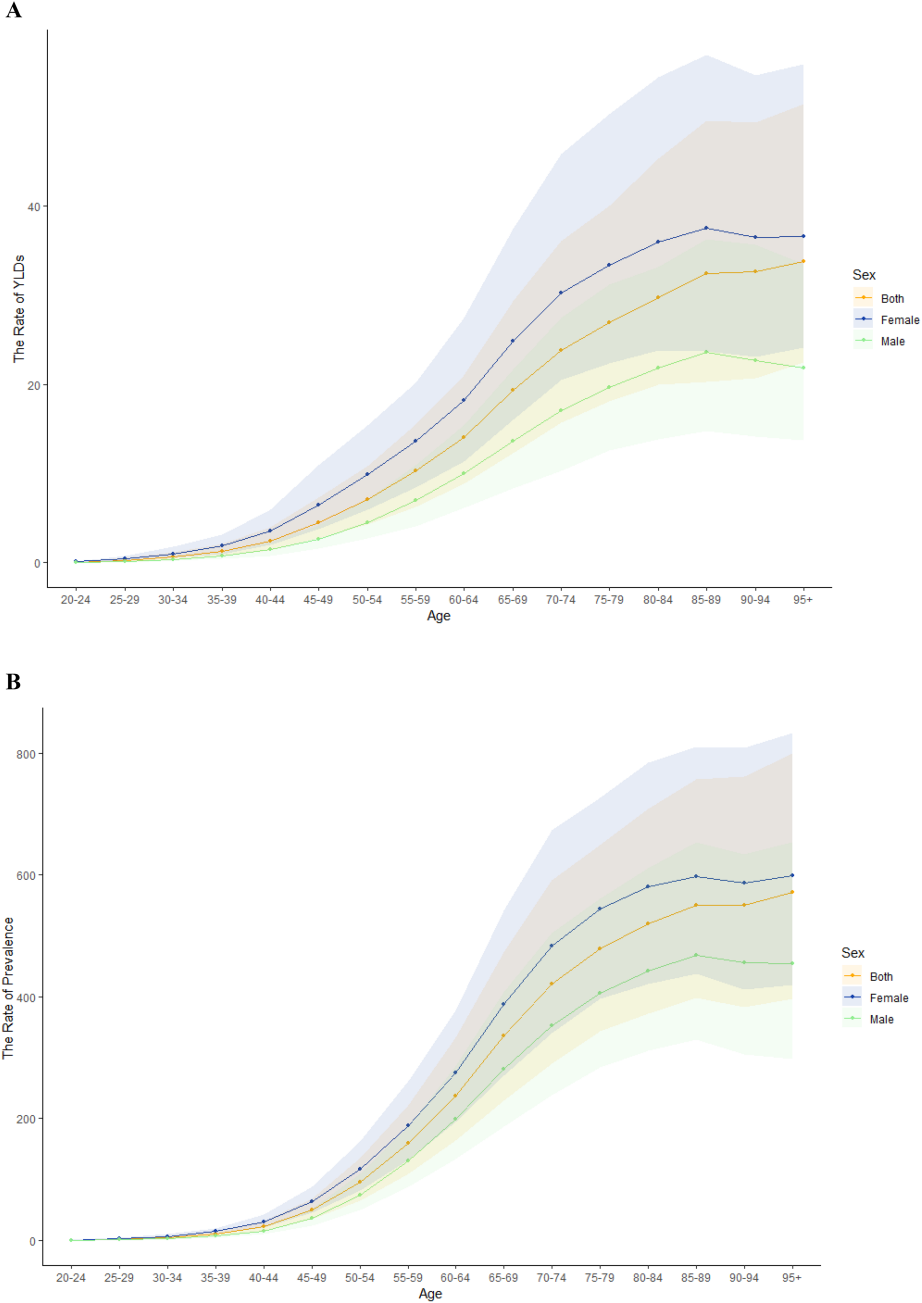
Age-standardized YLDs and prevalence rates of BVL due to DR in China in 2021. Age-standardized YLDs rate and prevalence rate of sex **(A, B)**.

The burden of BVL due to DR in China has undergone significant changes. **Figure 1** depicts the trends in ASYR and ASPR of DR in 2021. In China, the rates of YLDs and prevalence of DR showed a significant age-related increase. The disease burden was notably higher in women compared to men (**Figures 1A, B**). Regarding visual impairment severity, the burden of DR escalated markedly after age 50, peaking in individuals aged 95 years or older. Beyond age 60, the prevalence of MVL and the rate of YLDs attributable to DR surpassed those of SVL and blindness (**Supplementary Figures 1A, B**). Analysis by diabetes type revealed that T2DM was the predominant contributor to the DR disease burden (**Supplementary Figures 1C, D**).

**Figure 2:**
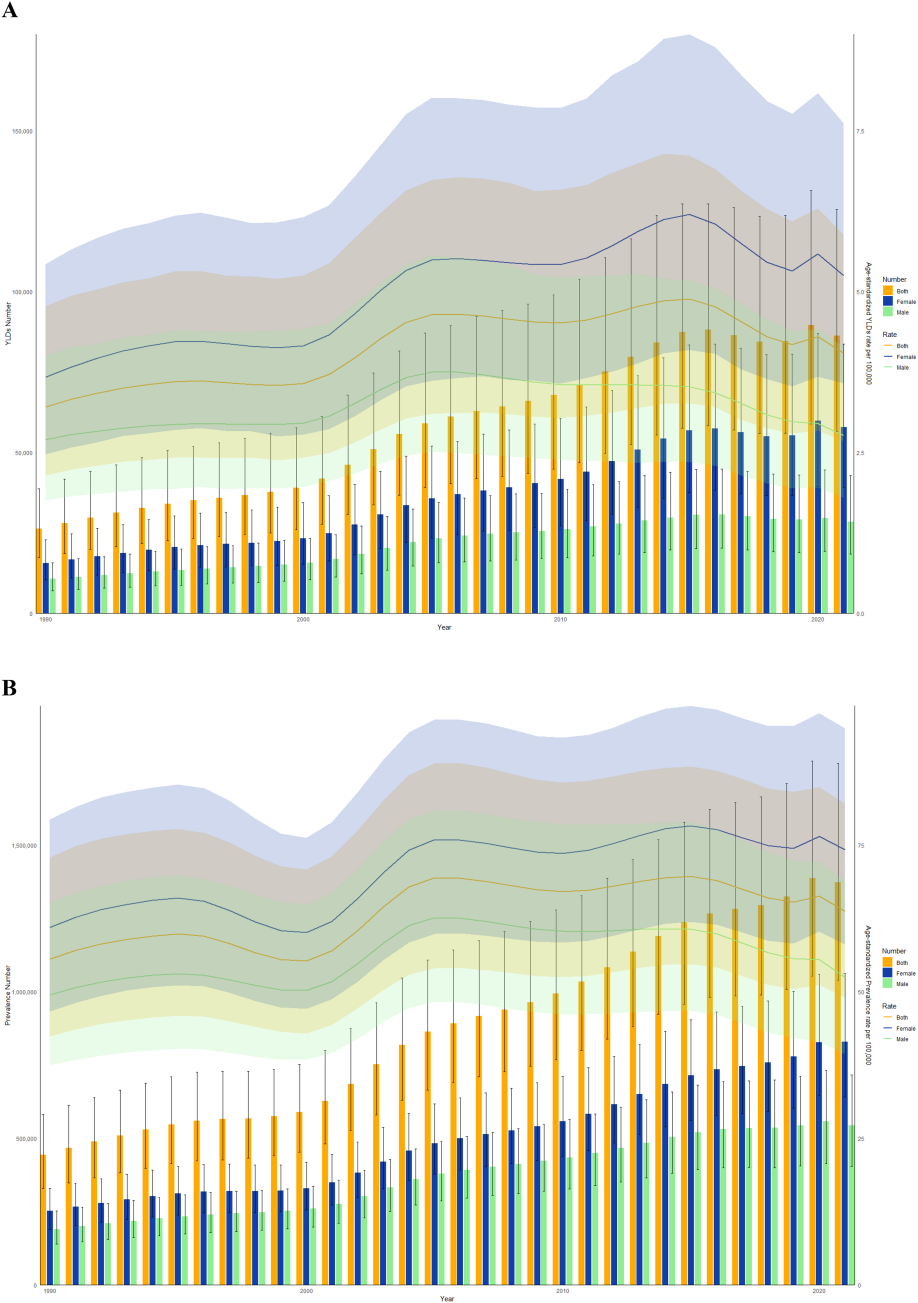
Trends in the all-age cases and age-standardized YLDs and prevalence rates of BVL due to DR by sex from 1990 to 2021 in China. **(A)** YLDs number and rate. **(B)** Prevalence number and rate.

From 1990 to 2021, the burden of disease caused by DR in both men and women in China demonstrated an overall upward trend (**Figure 2**). Over this period, the total number of all-ages YLDs due to DR rose significantly from 26,314 to 86,317, while the number of affected individuals across all age groups increased from 443,403 to 1,373,476. However, ASPR and ASYR of DR exhibited more nuanced trends, with a steady decline from 2015 to 2019, a rebound in 2020, and a subsequent decline in 2021. Notably, throughout the 32-year span, the disease burden of DR consistently remained higher among women compared to men. Further details on trends by levels of visual impairment and etiological subtypes are provided in **Supplementary Figure 2**.

### Joinpoint regression analysis

**Figure 3:**
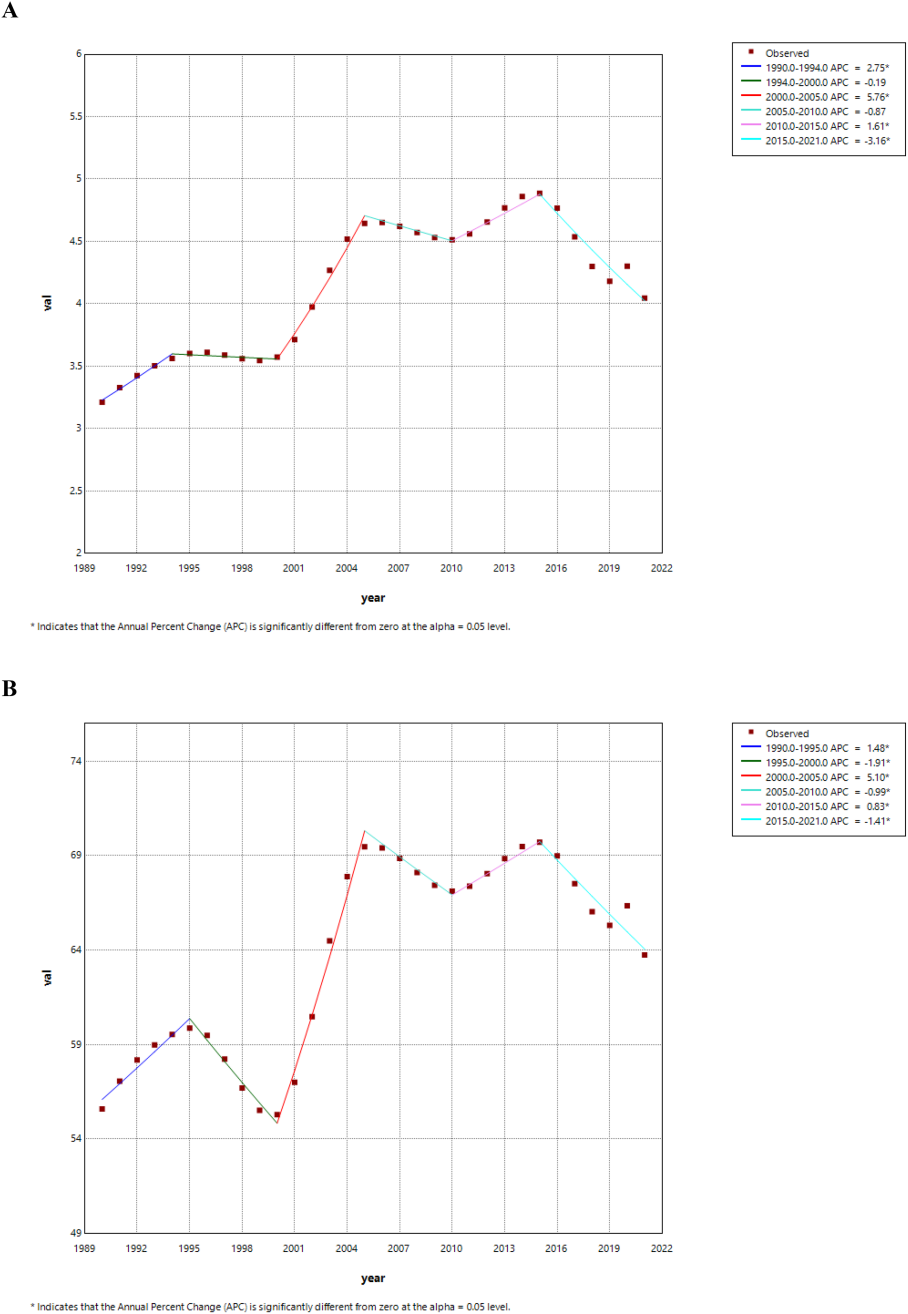
Joinpoint regression analysis of the age-standardized YLDs rate **(A)** and prevalence rate **(B)** for DR in China from 1990 to 2021.

**Figure 3** presents the joinpoint regression analyses of the ASYR and ASPR for DR in China during the time frame of 1990 to 2021. A pronounced increase was observed from 2000 to 2005 in both ASYR (APC = +5.76, 95% CI: 4.38, 7.16) and ASPR (APC = +5.10, 95% CI: 4.37, 5.84). Both ASYR (APC = -3.16, 95% CI: -3.84, -2.49) and ASPR (APC = -1.41, 95% CI: -1.78, -1.04) decline significantly from 2015 to 2021 (**Figures 3A, B**, **Table 2**). Over the span of 1990 to 2021, the AAPC for the ASYR and ASPR of DR in China rose by 0.71 (95% CI: 0.28, 1.14) and 0.43 (95% CI: 0.20, 0.66), respectively. Significantly, the analysis revealed that females, individuals with T1DM, and those with blindness demonstrated a more pronounced AAPC in both YLDs and prevalence rates, as compared to other demographic groups (**Supplementary Table 2**).

**Table 2:** Joinpoint regression analysis: trends in age-standardized YLDs and prevalence rates (per 100,000 persons) in China from 1990 to 2021.

ASYR			ASPR		
Period	APC(95%CI)	AAPC(95%CI)	Period	APC(95%CI)	AAPC(95%CI)
1990-1994	2.75* (1.39, 4.14)	0.71* (0.28, 1.14)	1990-1995	1.48* (0.94, 2.03)	0.43* (0.20, 0.66)
1994-2000	-0.19 (-1.12, 0.75)		1995-2000	-1.91* (-2.61, -1.20)	
2000-2005	5.76* (4.38, 7.16)		2000-2005	5.10* (4.37, 5.84)	
2005-2010	-0.87 (-2.15, 0.42)		2005-2010	-0.99* (-1.67, -0.29)	
2010-2015	1.61* (0.28, 2.95)		2010-2015	0.83* (0.15, 1.52)	
2015-2021	-3.16* (-3.84, -2.49)		2015-2021	-1.41* (-1.78, -1.04)	

APC, annual percent change; AAPC, average annual percent change presented for full period; CI, confidence interval; *P < 0.05.

### The effects of age, period, and cohort on prevalence rates

**Figure 4:**
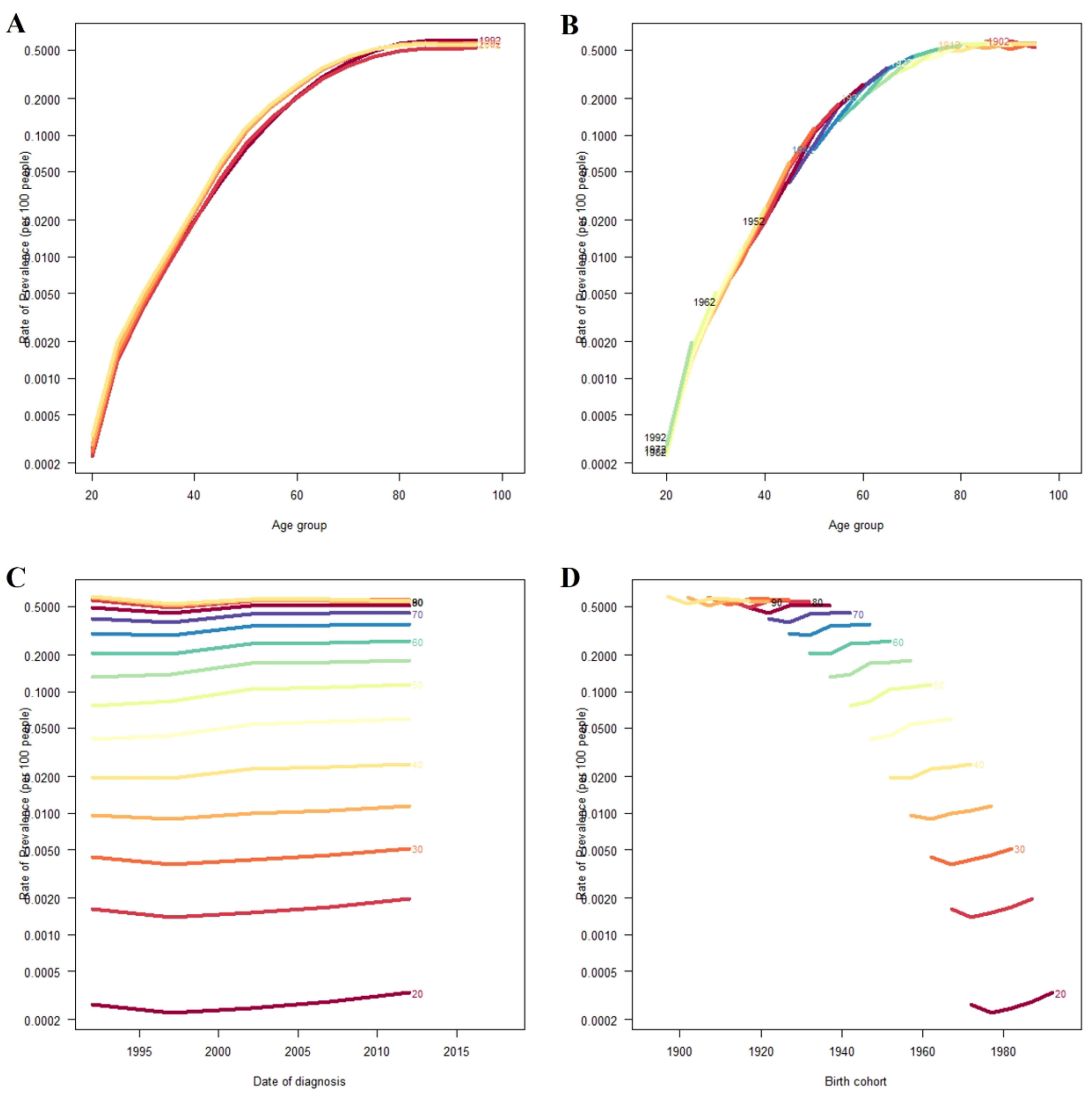
Overall prevalence rates of DR in China. **(A)** The age-specific prevalence rates of DR according to time periods; each line connects the age-specific prevalence for a 5-year period. **(B)** The age-specific prevalence rates of DR according to birth cohort; each line connects the age-specific prevalence for a 5-year cohort. **(C)** The period-specific prevalence rates of DR according to age groups; each line connects the date of diagnosis-specific prevalence for a 5-year age group. **(D)** The birth cohort-specific prevalence rates of DR according to age groups; each line connects the birth cohort-specific prevalence for a 5-year age group.

**Figure 4** illustrates the variations in age, period, and cohort effects on the overall prevalence rates of Chinese DR. In **Figure 4A**, the prevalence trends by age are presented for the years 1992, 1997, 2002, 2007, and 2012. The prevalence rate rose sharply between the ages of 40 and 60. **Figure 4B** displays the cohort trends in DR prevalence across various age groups. **Figure 4C** highlights the prevalence trends in the years between 1990 and 2021, showing a consistent increase over time across all age groups, with higher prevalence rates among older individuals. Finally, **Figure 4D** shows the changes in prevalence by cohort for specific age groups, indicating that DR prevalence rises with age in China.

### Decomposition analysis

**Figure 5:**
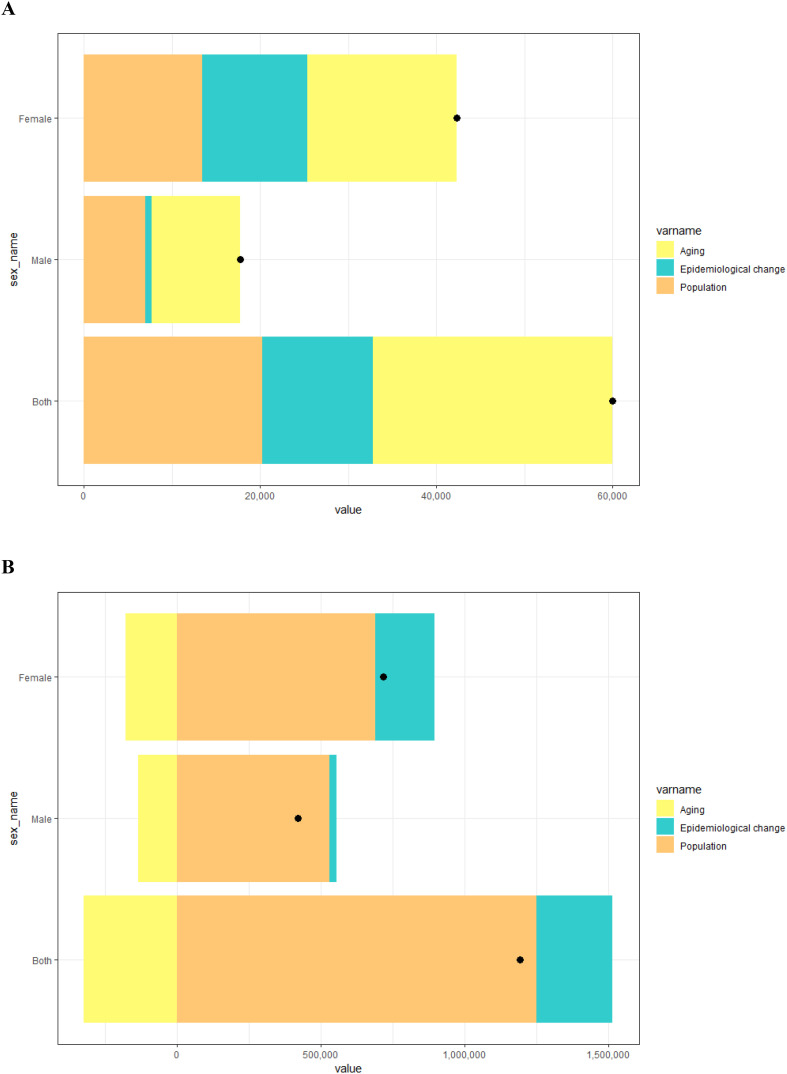
Decomposition analysis results for YLDs **(A)** and prevalence **(B)** of DR according to population-level determinants of population growth, aging, and epidemiological change from 1990 to 2021 in China. The black dot represents the overall value of change contributed by all three components.

The study conducted a decomposition analysis to quantify the impact of various factors influencing Chinese YLDs and prevalence of DR across the years 1990 to 2021. DR-related YLDs increased by 60,002.76 during this period, with the increase broken down into three factors: aging, epidemiological change, and population growth. Aging accounted for 27,229.80 (45.38%) of the increase, population growth for 20,192.66 (33.65%), and epidemiological change for 12580.30 (20.97%) (**Figure 5A**, **Supplementary Table 3**), indicating that aging has had the greatest impact on the disease burden in China. In terms of prevalence, there was an increase of 1.19 million cases covering the years 1990 through 2021. This increase was similarly decomposed into aging, population growth, and epidemiological change, contributing -322,015.19 (-27.02%), 1,249,953.30 (104.90%), and 263,644.29 (22.12%), respectively (**Figure 5B**, **Supplementary Table 3**). Population demographics are pivotal determinant in the prevalence of DR, with the aging process exerting a significant negative impact.

### BAPC analysis

**Figure 6:**
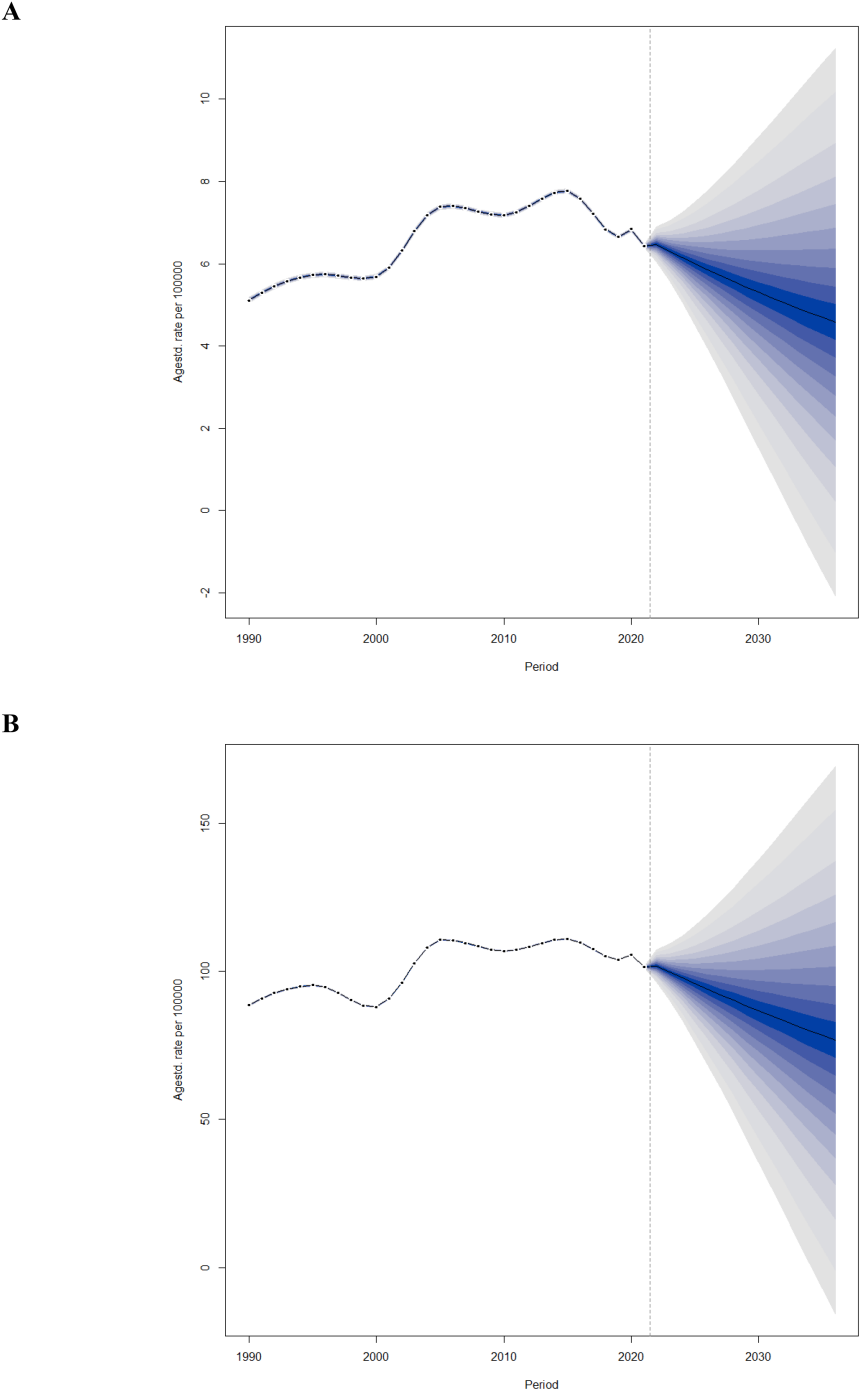
Trends of ASYR **(A)** and ASPR **(B)** of BVL due to DR in China from 2022 to 2036 predicted by Bayesian age-period-cohort models.

To examine the future trends of ASYR and ASPR for DR in China beyond 2021, we utilized BAPC models to project these rates from 2022 to 2036. As illustrated in **Figure 6A**, the ASYR is predicted to decrease annually from 6.47 per 100,000 in 2022 to 4.58 per 100,000 in 2036. **Figure 6B** illustrates that the ASPR will also decrease annually, from 101.76 per 100,000 in 2022 to 76.76 per 100,000 in 2036. Projections have been conducted to estimate the gender-specific disease burden of DR in China for the forthcoming decade and a half, delineating the anticipated trends for both male and female populations (**Supplementary Figure 3**).

## Discussion

DR remains a significant global public health concern and is one of the leading causes of visual impairment worldwide (6, 14). In China, the burden of DR has increased substantially over recent decades, driven by the growing prevalence of diabetes and demographic shifts. This study aimed to quantify the temporal trends and demographic influences on DR burden in the Chinese population based on data from the GBD 2021. Employing an innovative analytical framework, we examined trends in YLDs, prevalence, ASYR, and ASPR of DR spanning the period of 1990 to 2021. Our analysis revealed a steady increase in YLDs and DR cases over the 32-year period, peaking in 2015, followed by a decline that persisted after a brief rebound in 2020. Furthermore, projections for the next 15 years suggest that the growth of DR’s disease burden in China may moderate, influenced by the interplay of population aging and demographic shifts.

Our analysis revealed that the proportion of Chinese women affected by DR between 1990 and 2021 was significantly higher than that of men, consistent with previous studies (8). This gender disparity in DR burden was evident across all age groups. Several factors contribute to this difference. First, Chinese women generally have a longer life expectancy than men, increasing their cumulative risk of diabetes and related complications such as DR (15). Second, studies have shown that women, particularly in rural and economically disadvantaged regions, often have reduced access to healthcare services, including diabetic screening and ophthalmic examinations. For instance, a nationwide study reported lower diabetic retinopathy screening coverage among women compared to men in rural provinces (16). Third, biological and hormonal differences may also play a role. Declining estrogen levels during menopause have been associated with microvascular damage and may exacerbate DR progression. Furthermore, pregnancy is known to accelerate DR development in women with pre-existing diabetes, particularly during the second and third trimesters, due to metabolic and hemodynamic changes (17). Inadequate prenatal ophthalmic care may further elevate risks during pregnancy. In addition, dietary factors such as lower intake of retinal-protective nutrients (e.g., carotenoids, lutein) in women have also been linked to greater vulnerability to DR (18). These findings collectively underscore the need for gender-sensitive surveillance strategies, targeted screening programs, and health education initiatives that address the unique biological and social risk factors facing women with diabetes in China.

This study investigated the impact of aging on the disease burden of DR. In China, both the YLD rate and prevalence of DR in 2021 exhibited an increasing trend with advancing age, consistent with global findings identifying DR as a significant cause of visual impairment among middle-aged and older adults (19). While the ASYR and ASPR of DR have declined since peaking in 2015, a brief increase in 2020 interrupted this trend, followed by a continued decline. This improvement is largely attributed to initiatives such as the ‘Sight First China Action’, which has been instrumental in DR prevention and treatment efforts. Aging remains a critical risk factor for visual impairment (20). Our decomposition analysis highlights the adverse effects of population aging on DR prevalence. While the decomposition analysis illustrates the relative contributions of demographic and epidemiological factors to changes in DR burden, it is important to note that the direction and magnitude of aging’s effect may vary depending on the metric analyzed (e.g., total YLDs vs. age-standardized rates) and the reference year. For instance, population aging may contribute to an increase in the total number of cases (due to a growing elderly population), but simultaneously coincide with improvements in healthcare access or prevention that lower the age-standardized burden. These seemingly divergent effects reflect the multidimensional nature of disease burden metrics and do not indicate true contradictions. Rather, they highlight the importance of contextualizing each component within the broader demographic and epidemiologic framework. However, this does not suggest reducing prevention efforts for older adults; rather, it underscores the need for enhanced interventions, as vision loss in this population increases fall risk and overall mortality (21). Notably, while younger and middle-aged individuals experience lower DR prevalence and YLDs compared to older adults, the incidence of DR in younger populations is rising, driven by changes in dietary habits and lifestyle factors (22). To address this dual challenge, China should expand preventive healthcare services targeting the elderly while simultaneously strengthening DR screening programs for younger populations.

To further address the potential confounding effect of period and cohort factors on age-specific DR burden, we performed a formal age–period–cohort (APC) analysis using data from 1990 to 2021. The APC model revealed that the prevalence of diabetic retinopathy increased markedly with age, particularly between the ages of 40 and 60, which is consistent with our descriptive findings in **Figure 1**. However, the model also indicated that more recent cohorts and later periods experienced higher baseline prevalence levels across all age groups, suggesting that generational changes and evolving risk exposures may contribute to the observed patterns. These results validate the predominant role of age while also emphasizing that both period-related (e.g., diagnostic awareness, screening) and cohort-specific (e.g., lifestyle, environmental) factors have significant influence on DR prevalence trends in China. The inclusion of APC analysis thus strengthens the temporal interpretation of our findings.

It is worth noting that the epidemiological component in our decomposition analysis may partially reflect improvements in diabetes diagnosis rates and advancements in DR screening technologies, which have led to increased case detection over time. These changes are intertwined with broader healthcare system developments and public health initiatives. However, due to the aggregate nature of the GBD data, we were unable to disentangle the specific contribution of each confounder, such as improved medical access or diagnostic sensitivity. As such, the attribution of YLDs increase to demographic factors should be interpreted cautiously, and future studies incorporating more granular health service data and stratified modeling approaches are warranted to refine these estimates.

Blindness and visual impairment significantly diminish quality of life, adversely affecting both physical and mental health while reducing personal autonomy (23). Regarding visual impairment caused by DR, this study highlights that MVL constitutes a substantial proportion of cases. In recent years, ASYR and ASPR for SVL and blindness have shown a declining trend, likely reflecting advancements in DR medical technologies. To build on this progress, increased investment in DR treatment and timely intervention for MVL patients are essential to prevent disease progression to more severe stages. Additionally, this study confirms that T2DM remains the primary contributor to DR-related disease burden, consistent with prior findings (24). To address this, China should implement comprehensive policies focused on diabetes prevention, management of complications, promotion of physical activity, and improvements in dietary habits to enhance overall quality of life (25).

The number of DR cases and associated YLDs in China has shown an upward trend over most years. However, age-standardized rate data indicate a decline in the DR burden since 2016, largely due to global advancements in treatment technologies, such as improvements in vitrectomy and the widespread adoption of anti-vascular endothelial growth factor therapies (26). Projections for the next 15 years suggest that while the DR burden will likely continue to decrease, sustained vigilance is essential. To assess the reliability of our forecasts, we employed retrospective validation using the ‘retro=TRUE’ setting in the BAPC model, which generated predictions for previous years and compared them to observed data. The strong alignment between predicted and actual values supports the model’s validity. However, it should be noted that the model extrapolates based on historical trends and does not incorporate future factors such as the implementation of national diabetic retinopathy screening programs, advances in AI-assisted diagnostics, or healthcare policy reforms. These developments could lead to an even steeper decline in disease burden than currently projected. China must therefore strengthen efforts to prevent and mitigate current and emerging risk factors associated with DR to further reduce the disease burden and enhance the quality of life for affected individuals.

While the GBD 2021 dataset provides comprehensive and standardized global estimates, we acknowledge that there may be discrepancies between GBD-derived estimates and real-world epidemiological data in China. Several population-based and hospital-based studies in China have reported DR prevalence rates that differ from those presented in the GBD dataset. For instance, a national cross-sectional survey reported a DR prevalence of 27.9% among patients with type 2 diabetes in urban regions, while a rural cohort study showed significantly lower screening rates and delayed diagnoses (27). These discrepancies may result from variations in screening coverage, diagnostic criteria, and data completeness. Although a formal sensitivity analysis could not be conducted due to the lack of publicly available region-specific datasets with consistent definitions and metrics, our findings remain valid in the context of national-level burden estimation. Future work incorporating localized datasets is warranted to further refine regional estimates and validate GBD-based projections. Furthermore, the GBD modeling framework may not fully reflect the impact of national public health strategies and surveillance systems in countries like China. Over the past decade, China has implemented several initiatives, such as the “Sight First China Action” program and the integration of diabetic retinopathy screening into national noncommunicable disease management. Pilot projects utilizing AI-assisted retinal image screening have also been expanding in primary care settings. These interventions may have accelerated the detection and treatment of DR more effectively than reflected in global estimates. Hence, reliance solely on global models could overlook significant local progress, underscoring the need for future analyses to integrate both national policy impact and real-world implementation data.

In addition to differences between GBD and real-world estimates, it is also important to consider the potential impact of regional heterogeneity within China. The country exhibits pronounced geographic disparities in socioeconomic development, healthcare infrastructure, and accessibility of diabetic screening services. Urban and eastern regions typically benefit from more advanced medical systems and regular screening programs, whereas rural and western regions often face shortages of ophthalmic professionals and diagnostic facilities. For example, rural-based studies have reported lower DR detection rates and greater delays in diagnosis compared to urban areas (5, 28). Although the current GBD dataset does not include subnational estimates, acknowledging these disparities is critical for the interpretation of disease burden. Future studies incorporating provincial-level data would be valuable for informing region-specific strategies and health policy planning. Moreover, substantial inter-provincial variation may exist due to disparities in economic development, healthcare investment, and implementation of DR screening initiatives. Unfortunately, subnational data at the provincial level are not currently available from the GBD study, which limits our ability to perform more granular analysis. Future national burden estimations would benefit from incorporating province-level surveillance data to improve the accuracy and policy relevance of region-specific health planning.

A key strength of this study lies in its systematic analysis of the spatial and temporal distribution of the DR burden in China from 1990 to 2021. Utilizing the latest data from GBD 2021 and a standardized methodology, the study also provides projections for DR burden trends over the next 15 years. However, several limitations should be acknowledged. To begin with, the selection of data sources and statistical assumptions inherent in the GBD study may have caused an inclination toward bias in the estimation workflow. Furthermore, this analysis was limited to the national level, as epidemiological data from remote or rural areas in China may be less accurate, and region-specific details remain unavailable.

## Conclusion

In conclusion, the last 32 years have been characterized by a growing burden of DR in China, as evidenced by rising YLDs and prevalence rates. While projections indicate a potential decline in the overall DR burden over the next 15 years due to demographic shifts and aging trends, this study highlights that women and elderly populations remain particularly vulnerable to DR. To address this significant public health challenge, China must optimize resource allocation and healthcare services, with a focus on enhancing DR screening, prevention, and management strategies, as well as mitigating related complications. Targeted interventions for high-risk groups will be critical to effectively reduce the disease burden and improve population health outcomes.

## Data Availability

The original contributions presented in the study are included in the article/**Supplementary Material**. Further inquiries can be directed to the corresponding author.
