# Supplementary material for: Chinese burdens and trends of diabetic retinopathy 1990–2021 and 15 years forecast: results from the Global Burden of Disease Study 2021
Source: Front Endocrinol (Lausanne). 2025 May 13;16:1573581. doi: 10.3389/fendo.2025.1573581 (PMC12106008; doi:10.3389/fendo.2025.1573581)

**Supplementary Table 1** All-age cases and age-standardized YLDs, and prevalence rates in 1990 for DR in China.

| **Measure** | **Rei** | **All-ages cases (95%UI)** | | | **Age-standardized rates per 100000 people (95%UI)** | | |
| --- | --- | --- | --- | --- | --- | --- | --- |
|  |  | **T1DM** | **T2DM** | **DM** | **T1DM** | **T2DM** | **DM** |
| **YLDs** | **MVL** | 89 (44,163) | 10755 (5800,18734) | 10844 (5850,18904) | 0.01 (0.01,0.02) | 1.36 (0.74,2.35) | 1.37 (0.74,2.36) |
|  | **SVL** | 53 (28,93) | 5595  (3195,9527) | 5648  (3227,9606) | 0.01 (0,0.01) | 0.73 (0.43,1.21) | 0.73 (0.43,1.22) |
|  | **B** | 124 (68,206) | 9698 (5861,15184) | 9822 (5930,15401) | 0.01 (0.01,0.02) | 1.09 (0.67,1.7) | 1.11 (0.68,1.72) |
|  | **BVL** | 265 (162,404) | 26049 (17146,38365) | 26314 (17325,38743) | 0.03 (0.02,0.04) | 3.18 (2.12,4.73) | 3.21 (2.14,4.77) |
| **Prevalence** | **MVL** | 2875 (1898,4088) | 355143 (251730,478476) | 358018 (253384,482062) | 0.33 (0.22,0.48) | 45.03 (32.78,60.75) | 45.36 (33,61.22) |
|  | **SVL** | 285 (176,413) | 31306 (20642,44290) | 31591 (20844,44627) | 0.03 (0.02,0.05) | 4.11 (2.77,5.76) | 4.14 (2.79,5.8) |
|  | **B** | 659 (442,972) | 53135 (38616,72604) | 53794 (39115,73456) | 0.07 (0.04,0.09) | 6.03 (4.46,8.18) | 6.09 (4.51,8.26) |
|  | **BVL** | 3819 (2664,5281) | 439584 (326587,578230) | 443403 (329132,582701) | 0.43 (0.31,0.6) | 55.16 (42.09,72.37) | 55.59 (42.4,72.96) |

YLDs, years lived with disability; MVL, moderate vision loss; SVL, severe vision loss; B, blindness; BVL, blindness and vision loss; UI, uncertainty interval.

**Supplementary Figure 1**

Age-standardized YLDs and prevalence rates of BVL due to DR in China in 2021. Age-standardized YLDs rate and prevalence rate of type (A, B). Age-standardized YLDs rate and prevalence rate of cause (C, D).

**
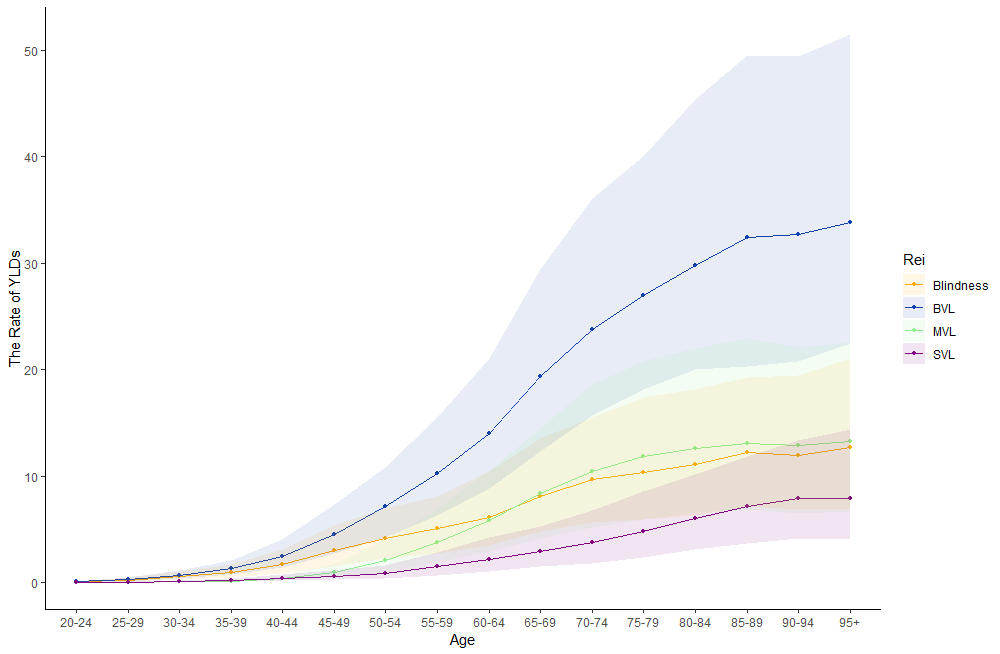
A**


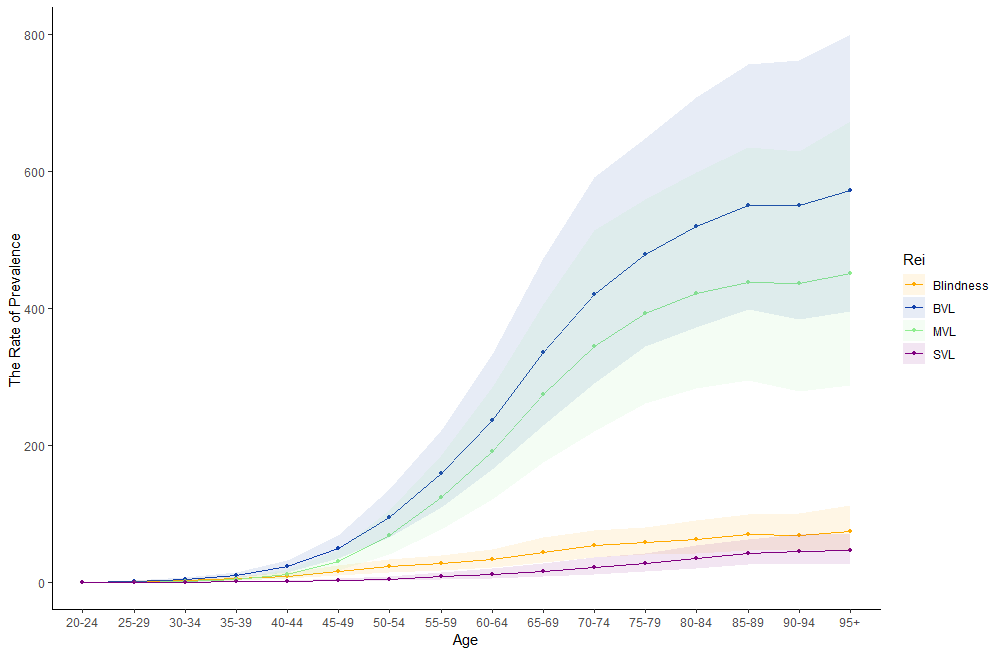


**B**

**
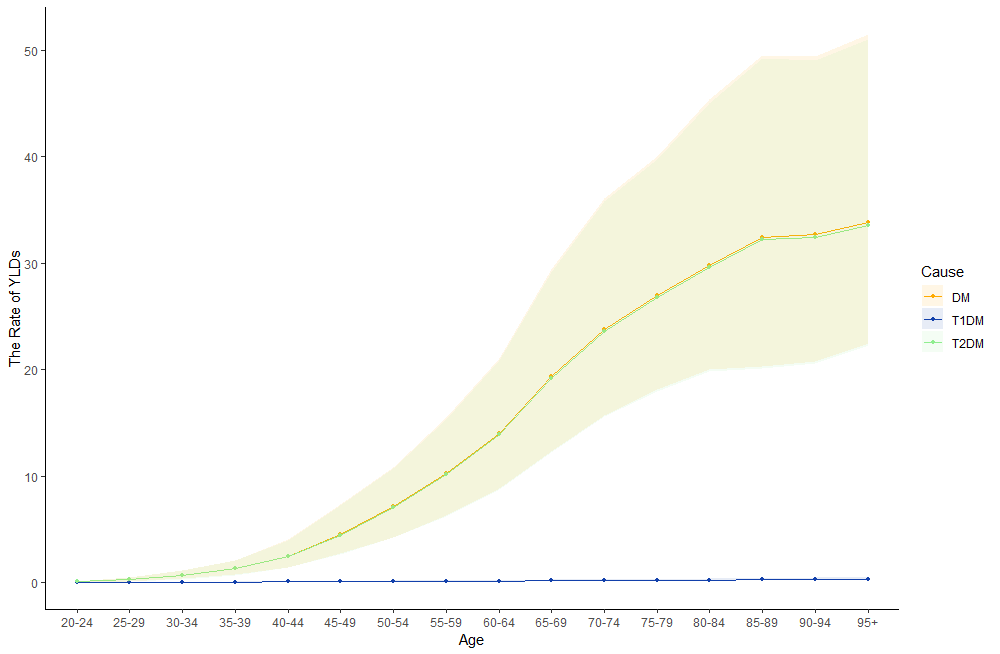
C**


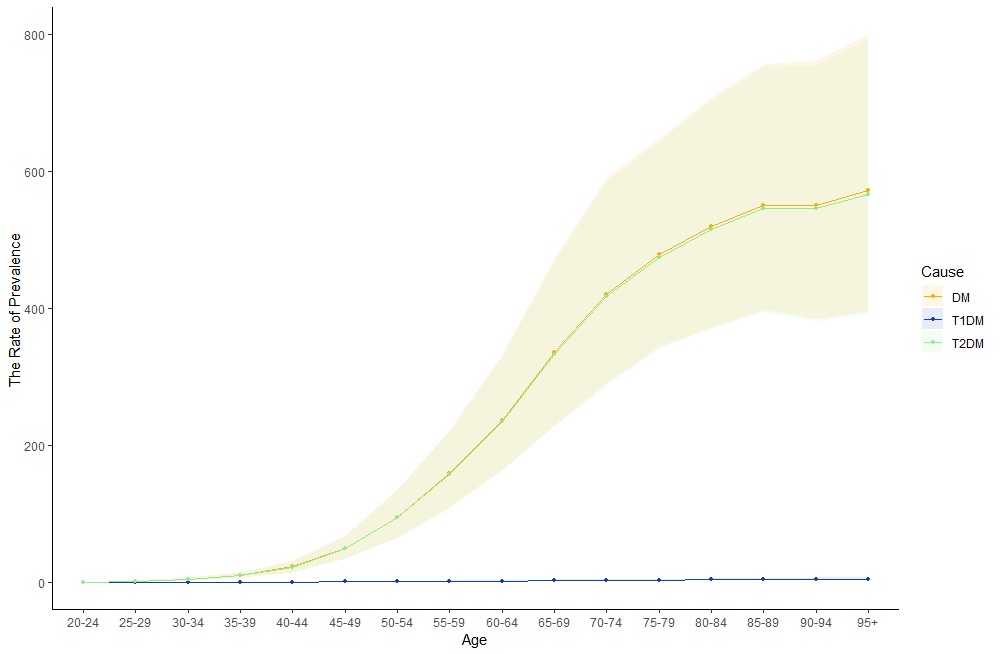


**D**

**Supplementary Figure 2**

Trends in the all-age cases and age-standardized YLDs and prevalence rates of BVL due to DR from 1990 to 2021 in China by type (A, B) and cause (C, D).


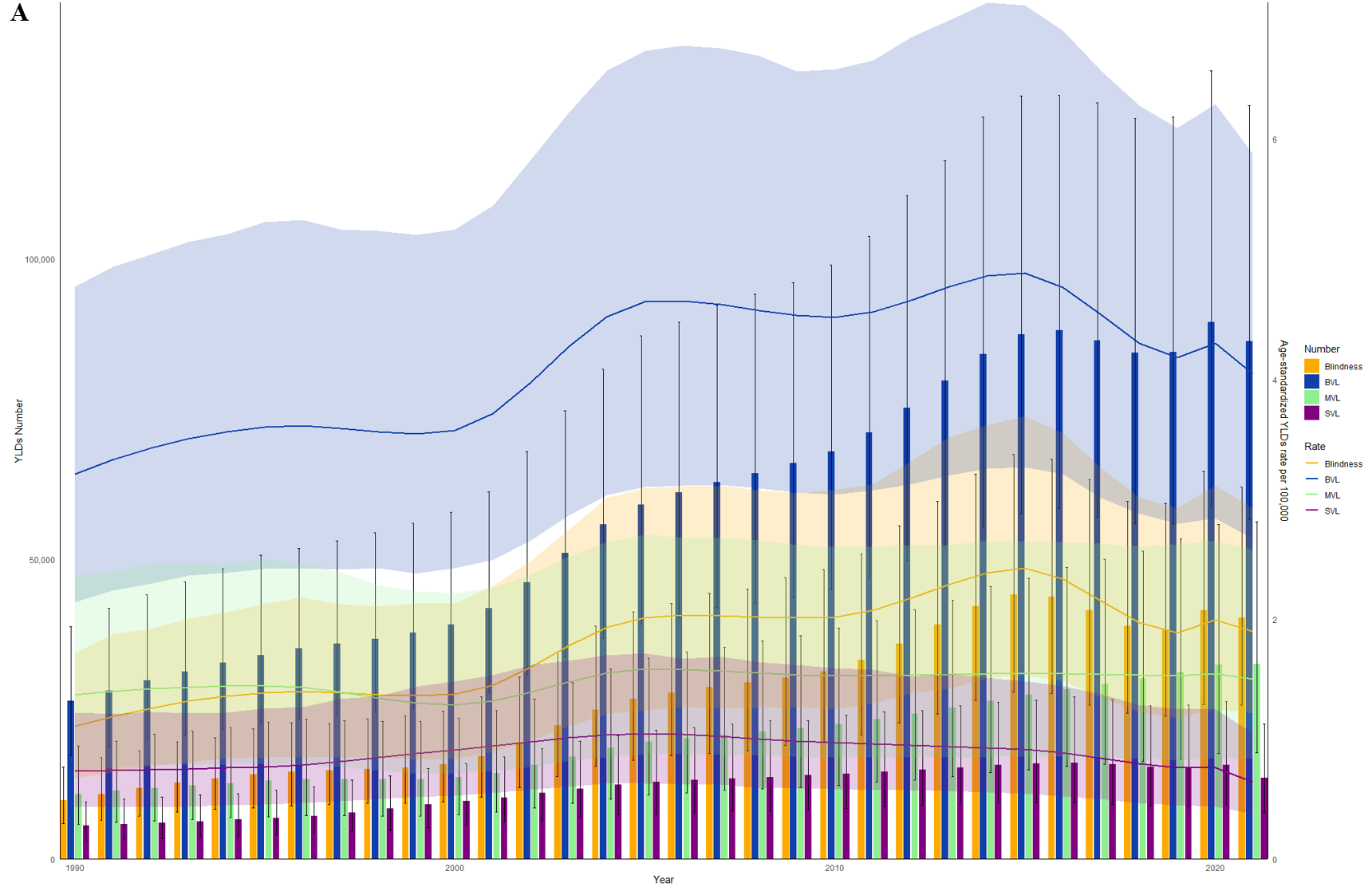


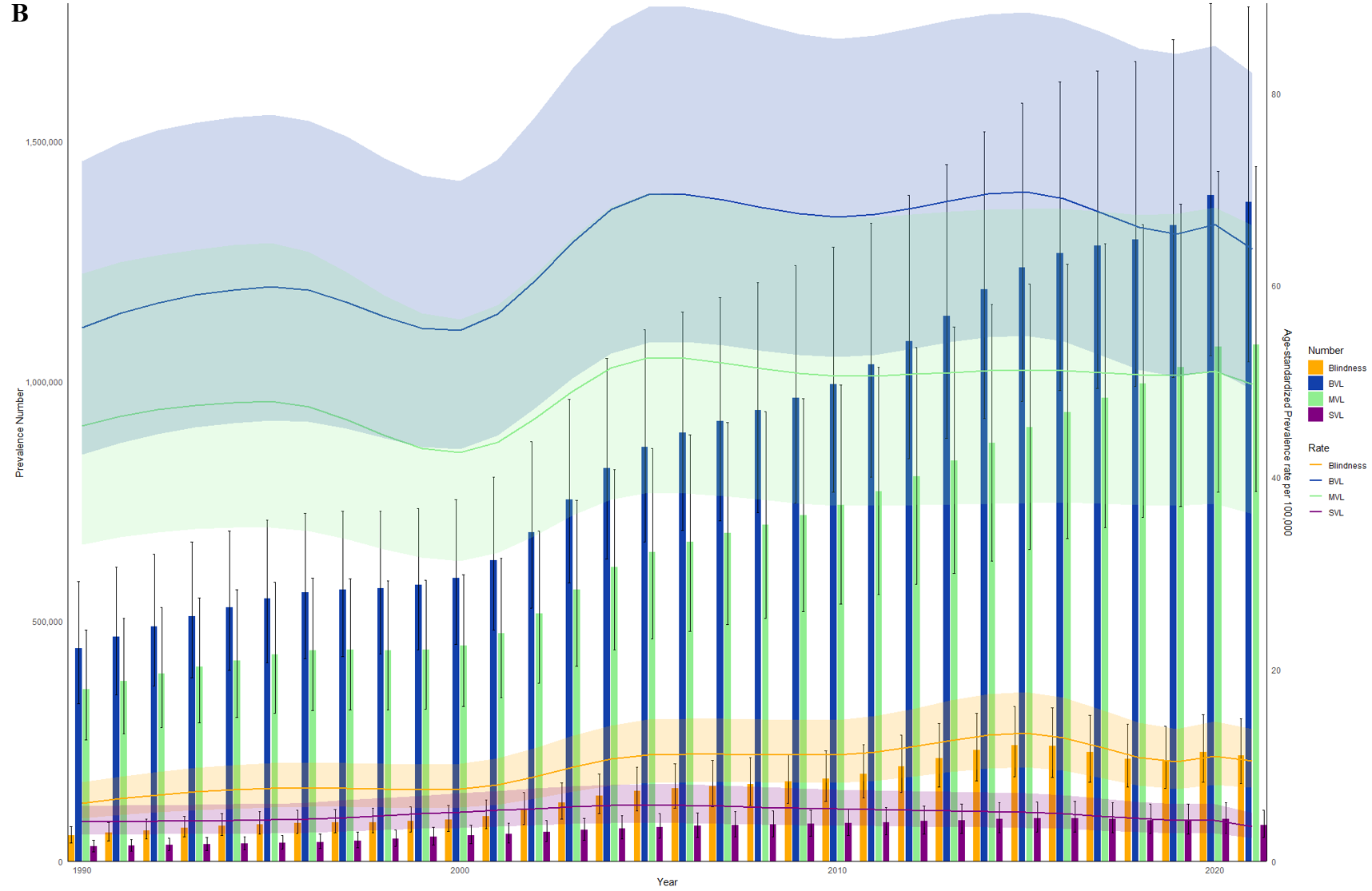


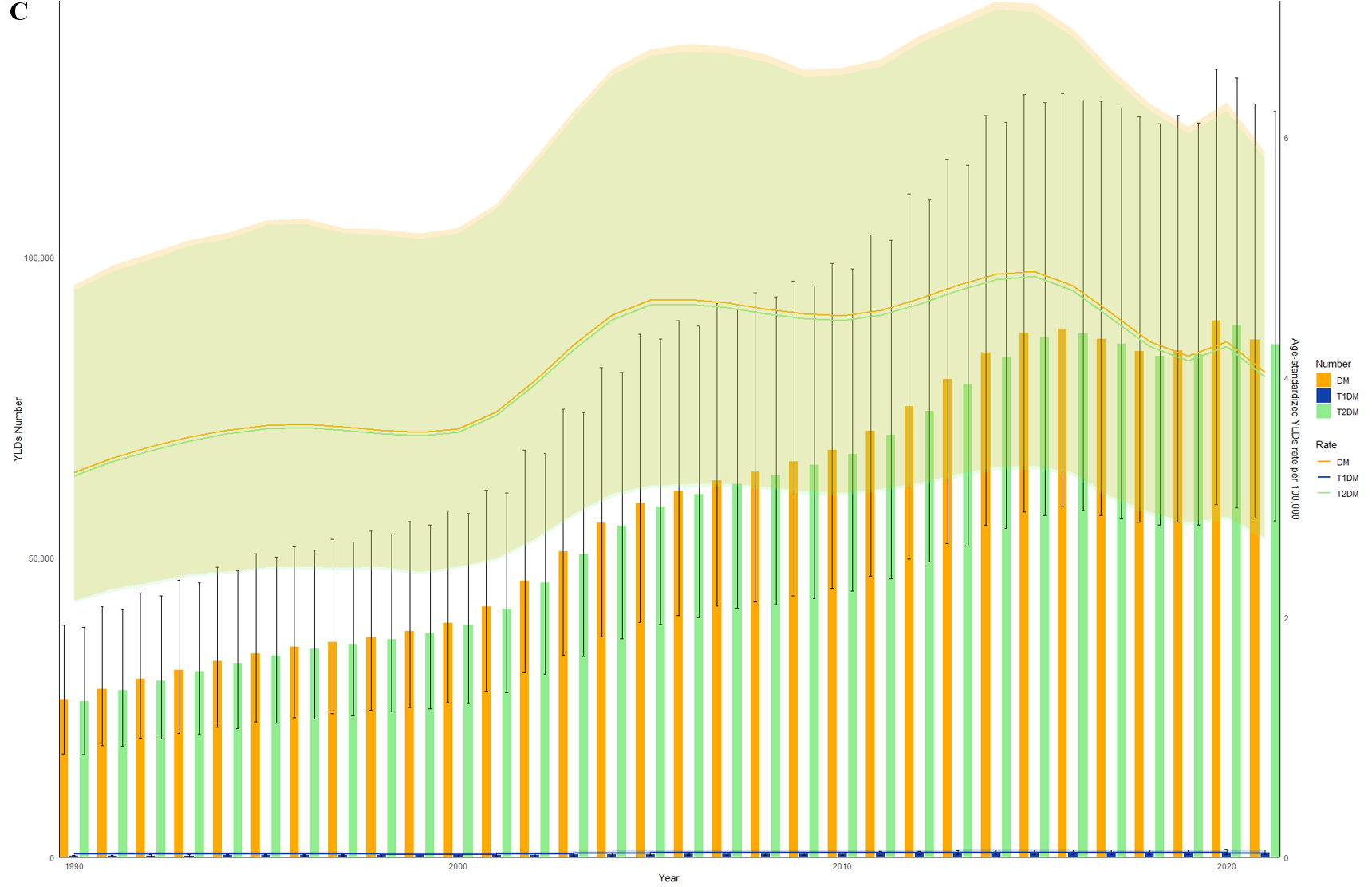


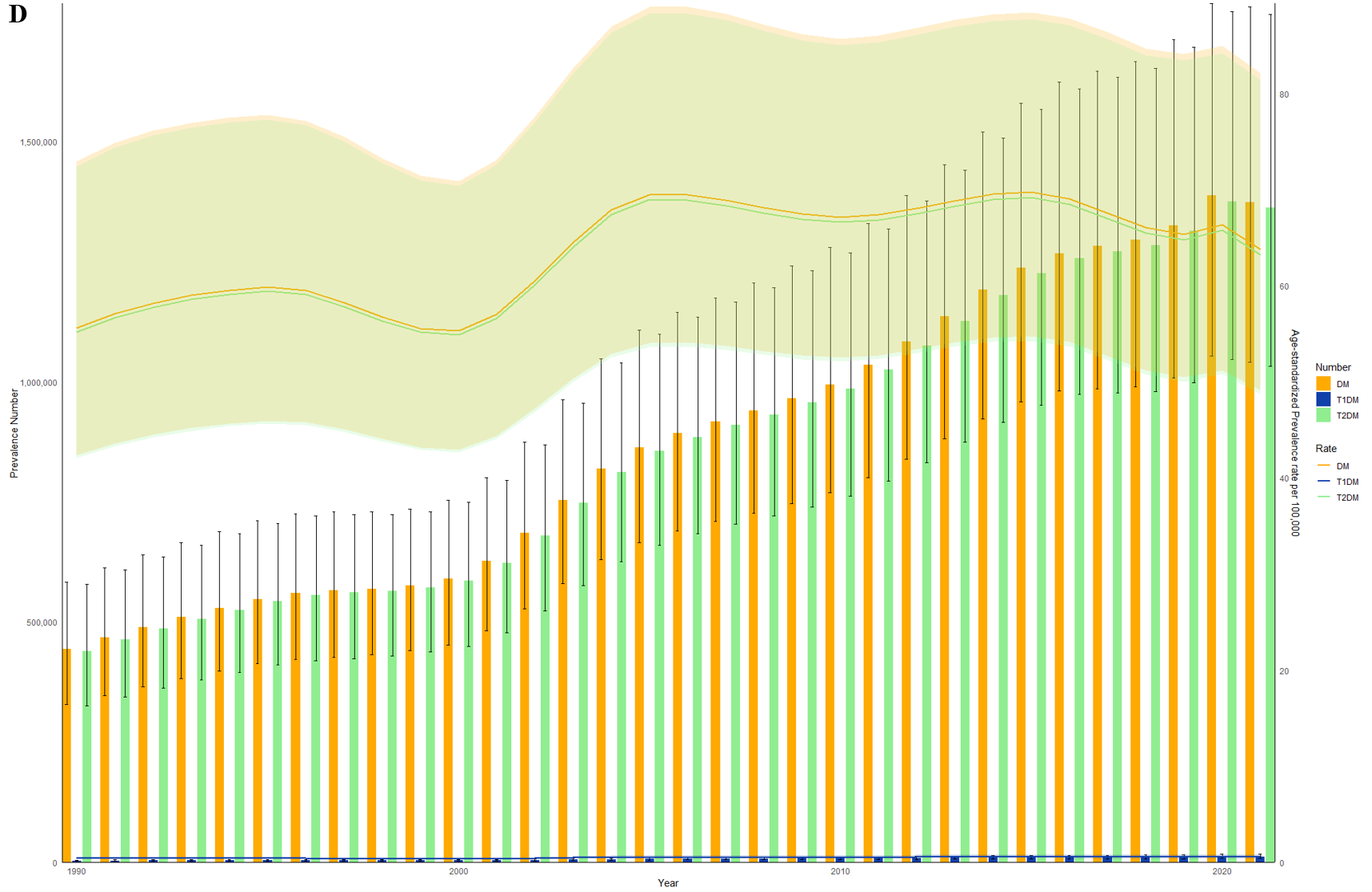


**Supplementary Table 2**

Joinpoint regression analysis: trends in age-standardized YLDs and prevalence rates (per 100,000 persons) among sexes, causes, and subtypes in China from1990 to 2021.

| **Type** | **ASYR** | | |  | **ASPR** | | |
| --- | --- | --- | --- | --- | --- | --- | --- |
|  | **Period** | **APC(95%CI)** | **AAPC(95%CI)** |  | **Period** | **APC(95%CI)** | **AAPC(95%CI)** |
| **Sex** |  |  |  |  |  |  |  |
| **Female** | 1990-1994 | 3.35* (1.62 , 5.11) | 1.14* (0.56 , 1.72) |  | 1990-1995 | 1.57* (0.94 , 2.22) | 0.61* (0.33 , 0.90) |
|  | 1994-2000 | -0.30 (-1.48 , 0.90) |  |  | 1995-2000 | -2.20* (-3.03 , -1.37) |  |
|  | 2000-2005 | 6.13* (4.35 , 7.94) |  |  | 2000-2005 | 5.24* (4.38 , 6.11) |  |
|  | 2005-2010 | -0.74 (-2.38 , 0.92) |  |  | 2005-2010 | -1.01* (-1.80 , -0.20) |  |
|  | 2010-2014 | 3.60* (0.90 , 6.37) |  |  | 2010-2014 | 1.65* (0.38 , 2.94) |  |
|  | 2014-2021 | -2.31* (-2.99 , -1.62) |  |  | 2014-2021 | -0.67* (-1.01 , -0.33) |  |
| **Male** | 1990-1994 | 1.98* (1.23 , 2.73) | 0.07 (-0.16 , 0.31) |  | 1990-1995 | 1.40* (0.92 , 1.89) | 0.21 (-0.01 , 0.42) |
|  | 1994-2000 | 0.06* (-0.46 , 0.59) |  |  | 1995-2000 | -1.39* (-2.03 , -0.74) |  |
|  | 2000-2005 | 5.27 (4.52 , 6.03) |  |  | 2000-2005 | 4.91* (4.25 , 5.58) |  |
|  | 2005-2010 | -1.37* (-2.08 , -0.66) |  |  | 2005-2010 | -1.05* (-1.68 , -0.41) |  |
|  | 2010-2015 | -0.11 (-0.83 , 0.61) |  |  | 2010-2015 | 0.27 (-0.38 , 0.92) |  |
|  | 2015-2021 | -3.94* (-4.32 , -3.57) |  |  | 2015-2021 | -2.25* (-2.59 , -1.90) |  |
| **Cause** |  |  |  |  |  |  |  |
| **T1DM** | 1990-1995 | 1.25 (-0.10 , 2.61) | 0.95* (0.38 - 1.52) |  | 1990-1995 | -0.58 (-1.54 , 0.39) | 0.92* (0.59 - 1.26) |
|  | 1995-2000 | -2.80* (-4.64 , -0.93) |  |  | 1995-2000 | -2.08* (-3.39 , -0.76) |  |
|  | 2000-2004 | 9.62* (6.35 , 12.99) |  |  | 2000-2005 | 7.72* (6.28 , 9.17) |  |
|  | 2004-2015 | 1.19* (0.71 , 1.67) |  |  | 2005-2021 | 0.30* (0.14 , 0.47) |  |
|  | 2015-2021 | -2.05* (-3.08 , -1.01) |  |  |  |  |  |
| **T2DM** | 1990-1994 | 2.76* (1.40 , 4.14) | 0.71* (0.28 - 1.14) |  | 1990-1995 | 1.50* (0.96 , 2.04) | 0.42* (0.19 - 0.66) |
|  | 1994-2000 | -0.17 (-1.10 , 0.76) |  |  | 1995-2000 | -1.91* (-2.61 , -1.20) |  |
|  | 2000-2005 | 5.74* (4.37 , 7.13) |  |  | 2000-2005 | 5.08* (4.35 , 5.82) |  |
|  | 2005-2010 | -0.88 (-2.15 , 0.41) |  |  | 2005-2010 | -0.99* (-1.68 , -0.30) |  |
|  | 2010-2015 | 1.60* (0.28 , 2.94) |  |  | 2010-2015 | 0.83* (0.15 , 1.51) |  |
|  | 2015-2021 | -3.17* (-3.84 , -2.50) |  |  | 2015-2021 | -1.42* (-1.79 , -1.05) |  |
| **Type** |  |  |  |  |  |  |  |
| **MVL** | 1990-1995 | 1.14* (0.68 , 1.60) | 0.34* (0.14 , 0.53) |  | 1990-1995 | 1.13* (0.67 , 1.60) | 0.33* (0.13 , 0.53) |
|  | 1995-2000 | -2.68* (-3.30 , -2.06) |  |  | 1995-2000 | -2.69* (-3.31 , -2.07) |  |
|  | 2000-2005 | 4.71* (4.05 , 5.37) |  |  | 2000-2005 | 4.70* (4.05 , 5.36) |  |
|  | 2005-2009 | -1.05* (-2.02 , -0.06) |  |  | 2005-2009 | -1.06* (-2.04 , -0.07) |  |
|  | 2009-2021 | -0.04 (-0.16 , 0.08) |  |  | 2009-2021 | -0.04 (-0.15 , 0.08) |  |
| **SVL** | 1990-1995 | 0.74 (-0.43 , 1.93) | -0.27 (-0.59 , 0.05) |  | 1990-1995 | 0.76 (-0.45 , 1.98) | -0.28 (-0.60 , 0.05) |
|  | 1995-2004 | 3.65* (3.07 , 4.24) |  |  | 1995-2004 | 3.64* (3.05 , 4.24) |  |
|  | 2004-2016 | -1.30* (-1.64 , -0.95) |  |  | 2004-2016 | -1.31* (-1.65 , -0.96) |  |
|  | 2016-2021 | -5.57* (-6.67 , -4.46) |  |  | 2016-2021 | -5.58* (-6.70 , -4.45) |  |
| **Blindness** | 1990-1994 | 5.50* (2.74 , 8.33) | 1.64* (0.73 , 2.55) |  | 1990-1994 | 5.54* (2.75 , 8.40) | 1.64* (0.74 , 2.55) |
|  | 1994-2000 | -0.24 (-2.12 , 1.68) |  |  | 1994-2000 | -0.23 (-2.08 , 1.65) |  |
|  | 2000-2005 | 8.48* (5.60 , 11.45) |  |  | 2000-2005 | 8.52* (5.73 , 11.38) |  |
|  | 2005-2010 | -0.62 (-3.14 , 1.96) |  |  | 2005-2010 | -0.65 (-3.19 , 1.95) |  |
|  | 2010-2014 | 5.04* (0.86 , 9.40) |  |  | 2010-2014 | 5.04* (0.85 , 9.42) |  |
|  | 2014-2021 | -3.76* (-4.80 , -2.71) |  |  | 2014-2021 | -3.77* (-4.82 , -2.71) |  |

APC, annual percent change; AAPC, average annual percent change presented for full period; CI, confidence interval; **P* < 0.05.

**Supplementary Table 3**

Changes in YLDs and prevalence number according to population-level determinants and causes from 1990 to 2021.

| **Sex** | **Overll difference** | **Changes in YLDs number due to Population-level determinants (% contribute to the total changes)** | | |  | **Overll difference** | **Changes in prevalence number due to Population-level determinants (% contribute to the total changes)** | | |
| --- | --- | --- | --- | --- | --- | --- | --- | --- | --- |
|  |  | **Aging** | **Population** | **epidemiological change** |  |  | **Aging** | **Population** | **epidemiological change** |
| Both | 60002.76 | 27229.80  (45.38%) | 20192.66  (33.65%) | 12580.30  (20.97%) |  | 1191582.1 | -322015.49  (-27.02%) | 1249953.30 (104.90%) | 263644.29 (22.12%) |
| Male | 17719.84 | 10079.84  (56.88%) | 6980.66  (39.39%) | 659.42  (3.73%) |  | 420560.67 | -134367.71  (-31.95%) | 531109.35 (126.29%) | 23819.03 (5.66%) |
| Female | 42282.92 | 16924.03  (40.03%) | 13445.44  (31.80%) | 11913.45  (28.17%) |  | 718065.67 | -178241.92  (-24.82%) | 688231.23 (95.85%) | 208076.36 (28.97%) |

**Supplementary Figure 3**

Trends of ASYR and ASPR of BVL due to DR in China from 2022 to 2036 in females (A,B) and males (C,D) predicted by Bayesian age-period-cohort models.


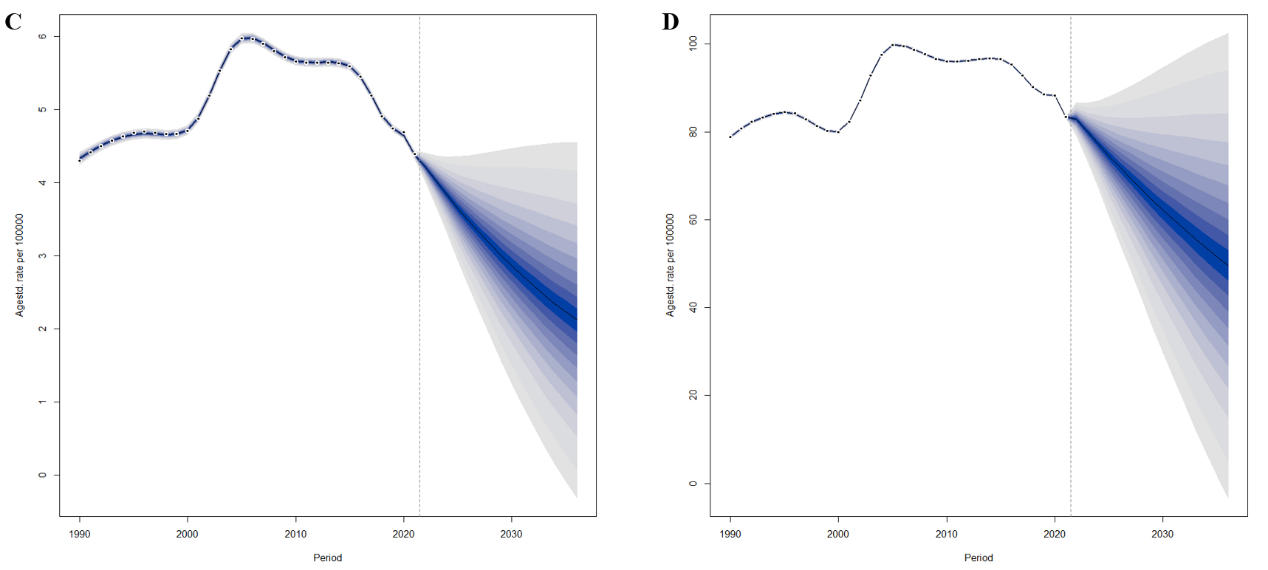

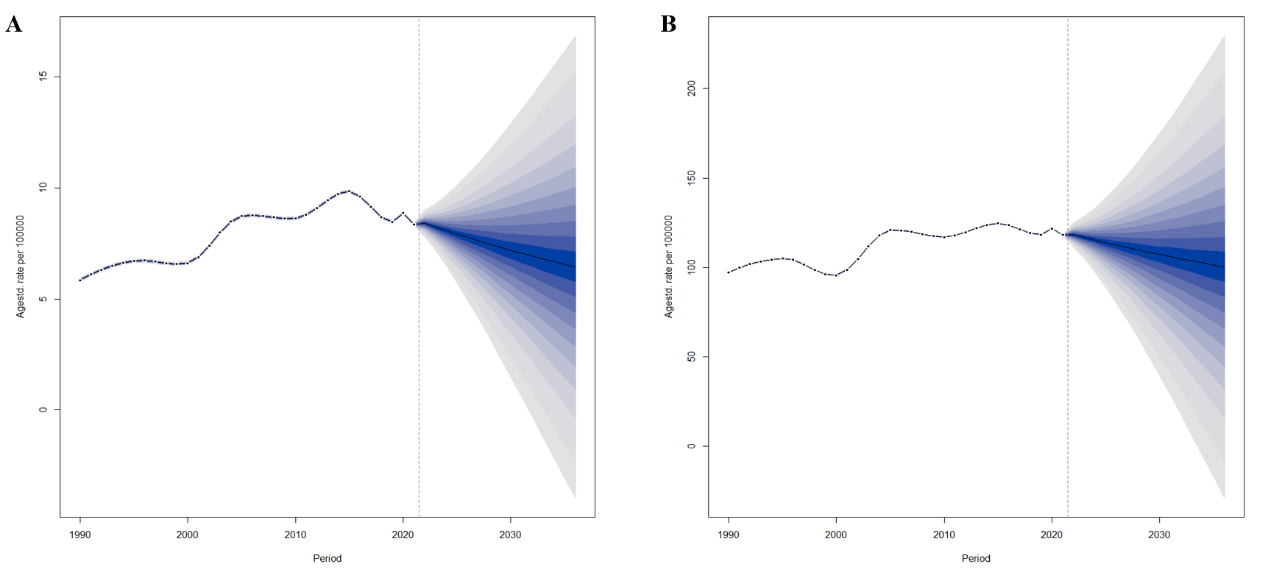

Supplement: Supplementary file 1 [file DataSheet1.docx]
